# Current utilization and influencing factors of complementary and alternative medicine among children with neuropsychiatric disease: a cross-sectional survey in Korea

**DOI:** 10.1186/s12906-016-1066-4

**Published:** 2016-03-01

**Authors:** Min-Jeong Jeong, Hye-Yoon Lee, Jung-Hwa Lim, Young-Ju Yun

**Affiliations:** Department of Pediatrics, College of Korean Medicine, Useok University, Jeonju, Republic of Korea; Department of Internal Medicine, Pusan National University Korean Medicine Hospital, Yangsan, Republic of Korea; Department of Neuropsychiatry, School of Korean Medicine, Pusan National University, Yangsan, Republic of Korea; Department of Integrative Medicine, School of Korean Medicine, Pusan National University, Yangsan, Republic of Korea

**Keywords:** Complementary and alternative medicine (CAM), Neuropsychiatric disease, Traditional Korean medicine

## Abstract

**Background:**

Complementary and alternative medicine (CAM) is widespread but has various utilization rates according to country and the condition of patients. Generally, CAM is more frequently used in diseases that have no clear treatment method in conventional medicine. Therefore, a high utilization rate of CAM can be assumed in pediatric neurological diseases, but few studies have investigated the utilization of CAM in children with neuropsychiatric diseases. In particular, studies regarding the current use of CAM are scarce.

**Methods:**

We conducted a survey of the parents or caregivers of patients who visited the pediatric rehabilitation clinic, pediatric neurology clinic, or pediatric psychiatry clinic at one university hospital from April to July 2011. We analyzed the factors that affect the utilization of CAM and other rehabilitation therapies.

**Results:**

Among the 578 patients recruited, 258 patients have ever received CAM (51.5 %), and the current CAM utilization rate was 19.0 % (110 patients). Two hundred patients (34.6 %) were currently receiving only other rehabilitation therapies, and 268 patients (46.4 %) were currently receiving no type of therapy. The rate of current CAM usage was significantly high in epilepsy patients.

The ORs of 1–6-year-old and 7–12-year-old children compared with 13–19-year-old children were 3.14 (95 % CI 1.31–7.53) and 3.34 (95 % CI 1.64–6.79), respectively, and the OR of the group with longer disease duration (≥48 months) compared with the group with shorter disease duration was 3.36 (95 % CI 1.71–6.59). Only the age and disease duration showed statistically significant differences between the patients who were administered CAM and those who received other rehabilitation therapies (*p* < 0.0001).

**Conclusions:**

CAM is preferred by patients under 13 years of age compared with patients aged 13–19 years, whereas other rehabilitation therapies are preferred by patients aged 1–6 years, followed by those aged 6–12 years and then by those aged 13–19 years. The patient’s age and disease duration are the major factors influencing CAM use. Future studies should specify particular diseases, rather than combining all types of neuropsychiatric diseases, and include the socio-economic status of the parents.

**Electronic supplementary material:**

The online version of this article (doi:10.1186/s12906-016-1066-4) contains supplementary material, which is available to authorized users.

## Background

Complementary and alternative medicine (CAM) is defined as “a broad domain of healing resources that encompasses all health systems, modalities, and practices, and their accompanying theories and beliefs, other than those intrinsic to the politically dominant health systems of a particular society or culture in a given historical period” [1]. CAM is used worldwide [2–6], and many national surveys have proven that CAM is widespread [7]. In certain African or Asian countries, CAM is used as the primary method of health care in the form of traditional medicine [8].

Some population-based studies have shown that 1/3 to 2/3 of the subjects were using CAM [4, 5]; however, population-based studies on children are scarce. According to a study conducted in the USA based on the 1996 Medical Expenditure Panel Survey database, pediatric CAM use is reported to be as low as 1.8–2.0 % [9, 10]. However, previous studies conducted in other countries found that the utilization rate ranged from a minimum of 13 % to a maximum of 80 % [4]. These were either hospital-based studies or studies that focused on children with specific diseases. A higher use of CAM was found in severe intractable diseases, such as tumors, or neurological diseases such as cerebral palsy (CP) [11–15]. A Korean study demonstrated that 35 % of school-aged children in the general population have used CAM [16], and in another study, 63.5 % of the children with a chronic disease were using CAM [17].

A high utilization rate of CAM can be assumed with pediatric neurological diseases because these conditions are usually chronic and intractable, but few studies have investigated this assumption. A study on the use of CAM in children with CP in the USA reported that 56 % of the subjects had ever received CAM [18]. A study that was performed in Canada in 2005 reported that 44 % of the children with neurological disorders had received CAM [11], and a study in Israel reported that 7.5 % of the children with attention deficit hyperactivity disorder (ADHD) and 14 % of the children with epilepsy were currently using CAM and that 51.5 % of the children with epilepsy had ever undergone treatment with CAM [19]. A Korean study on pediatric epilepsy reported that 17.2 % of the subjects had ever taken traditional Korean herbal medicine as a CAM approach [20].

In Korea, previous studies have focused on the use of traditional Korean herbal medicine and acupuncture, and research on the general use of CAM is lacking. Additionally, a past experience with CAM was surveyed [21, 22], but a study on the current use is still required. This study was conducted to investigate the current use of CAM in Korea in children with neurological or psychiatric diseases and the factors that affect the use of CAM.

## Methods

### Methods

A survey was conducted of the parents or caregivers of patients who visited the pediatric rehabilitation clinic (Additional file 1), pediatric neurology clinic or pediatric psychiatric clinic at one university hospital between April and July, 2011. After this study was approved by the Institutional Review Board, we explained the study to all the parents and caregivers, and those who signed the informed consent form were included. Patients under 1 or over 20 years of age were excluded. The guardians completed a questionnaire after listening to a detailed description by an interviewer. The self-reported questionnaire consisted of 12 multiple-choice questions, and the guardians were instructed to report the patient’s primary diagnosis, date of diagnosis, health problems other than the diagnosed disease, use of CAM in the past or present, and use of other rehabilitation therapies not included in CAM. “CAM use” was defined as the use of dietary supplements that do not need a doctor’s prescription, acupuncture therapy, traditional herbal medicine, neurofeedback, massage and other such therapies according to the definition and general situation reported by the WHO [23] and the National Center for Complementary and Alternative Medicine (NCCAM) [24]. “Other rehabilitation therapies” that were not included in CAM were defined as therapies that are prescribed or recommended by a doctor, such as physical therapy, occupational therapy, speech therapy, educational therapy (art, music, play), psychological counseling and cognitive behavioral therapy.

### Statistical analysis

The general and clinical characteristics of the subjects were summarized using frequencies and percentages for categorical data and means ± standard deviation and median (range) for continuous data.

For categorical data, the Chi-square test was used to analyze the correlation between the subject characteristics and CAM use, and for continuous data, a *t*-test or analysis of variance (ANOVA) was used. The Tukey-Kramer test was used for a post hoc analysis after the ANOVA.

To determine the variables that affect the use of CAM, univariate and multivariate multinomial logistic regression analyses were used. Additionally, the adjusted odds ratio (OR) and 95 % confidence interval (CI) were calculated using a multivariate logistic regression analysis, which considers several covariates simultaneously. The statistical significance was set below 0.05, and the SPSS 18.0 software was used for the analysis.

## Results

A total of 596 copies of questionnaires were collected, and 18 were excluded from the analysis because of ambiguous answers; 578 questionnaires were thus finally analyzed. The number of patients who had ever received CAM was 258 (51.5 %). As presented in Table 1, 110 patients were currently using CAM with or without other rehabilitation therapies (19.0 %). Regarding the type of CAM, 72 patients (12.5 %) reported the use of dietary supplements, and 24 (4.1 %) used traditional Korean herbal medicine, while 13 (2.2 %) used acupuncture. There were 200 subjects who were currently receiving other rehabilitation therapies only (34.6 %), and 268 were currently receiving neither of the treatments (46.4 %). Among the patients, 328 out of 578 were boys (56.7 %), and 250 were girls (43.3 %); there was a significant difference in the sex ratios among the three groups (*P* < 0.05). The mean age of the patients was 8.9 ± 4.9 years, and 227 patients (39.3 %) were in elementary school, which was the highest rate (39.3 %). The mean age of the group currently using CAM was 8.6 ± 4.0 years, and 54 subjects (49.1 %) were elementary school students (7–12 years old). There was a significant difference in the mean ages among the three groups (*P* < 0.0001).

**Table 1:** Patient characteristics

Variable	Overall	CAM			*P* value
		Current user (with or without other rehabilitation therapies)	Non-user with other rehabilitation therapies	Non-user without other rehabilitation therapies	
All participants	578 (100.0)	110 (19.0)	200 (34.6)	268 (46.4)	
Sex					
Male	328 (56.7)	55 (50.0)	127 (63.5)	146 (54.5)	0.042
Female	250 (43.3)	55 (50.0)	73 (36.5)	122 (45.5)	
Age (years)					
Mean ± SD	8.9 ± 4.9	8.6 ± 4.0a^a^	7.3 ± 4.5b	9.9 ± 5.2c	<0.0001
Median (range)	9 (1–19)	9 (1–19)	6 (1–19)	10 (1–19)	
Age (years)					
1–6 years (preschooler)	206 (35.6)	36 (32.7)	101 (50.5)	69 (25.7)	<0.0001
7–12 years (primary school child)	227 (39.3)	54 (49.1)	69 (34.5)	104 (38.8)	
13–19 years (middle and high school child)	145 (25.1)	20 (18.2)	30 (15.0)	95 (35.4)	

^a^Differences in numeric characteristics between groups were tested with an analysis of variance (ANOVA) with the Tukey-Kramer post-hoc test. Means with different scripts are different from each other (*P* < 0.05)

Table 2 contains the patients’ clinical characteristics. Epilepsy was the most frequent diagnosis (67.3 %) and the primary diagnosis that was associated with the use of CAM or other rehabilitation therapies (*P* < 0.0001). The rate of current CAM usage was significantly high in epilepsy patients. The rate of receiving other rehabilitation therapies without CAM was significantly high in CP patients.

**Table 2:** Clinical condition of patients

Variable	Overall	CAM			*P* value
		Current user (with or without other rehabilitation therapies)	Non-user with other rehabilitation therapies	Non-user without other rehabilitation therapies	
Primary Diagnosis					
Epilepsy	389 (67.3)	85 (77.3)	101 (50.5)	203 (75.7)	<0.0001
CP	56 (9.7)	9 (8.2)	41 (20.5)	6 (2.3)	
Developmental disorder^a^	51 (8.8)	6 (5.5)	27 (13.5)	18 (6.7)	
ADHD	43 (7.4)	7 (6.4)	11 (5.5)	25 (9.3)	
Others	39 (6.7)	3 (2.7)	20 (10.0)	16 (6.0)	
Disease Duration (mos)					
Mean ± SD	62.1 ± 47.6	65.5 ± 43.9ab	68.3 ± 46.5a	55.5 ± 49.4b	0.015
Median (range)	48 (0–229)	57 (1–211)	55 (0–229)	38 (0–226)	
Disease Duration					
< 48 months	137 (33.9)	20 (25.6)	37 (24.2)	80 (46.2)	<0.0001
≥ 48 months	267 (66.1)	58 (74.4)	116 (75.8)	93 (53.8)	
Having Health Problems					
Yes	341 (59.0)	67 (60.9)	132 (66.0)	142 (53.0)	0.016
No	237 (41.0)	43 (39.1)	68 (34.0)	126 (47.0)	
Number of Health Problems					
Mean ± SD	0.8 ± 0.9	0.9 ± 0.9ab	0.9 ± 0.9a	0.7 ± 0.8b	0.007
Median (range)	1 (0–5)	1 (0–4)	1 (0–5)	1 (0–4)	
Health Problems					
Atopic dermatitis	60 (10.4)	10 (9.1)	20 (10.0)	30 (11.2)	0.81
Allergic rhinitis	103 (17.8)	21 (19.1)	27 (13.5)	55 (20.5)	0.14
Asthma	19 (3.3)	1 (0.9)	9 (4.5)	9 (3.4)	0.34
Dyspepsia	40 (0.9)	8 (7.3)	16 (8.0)	16 (6.0)	0.68
Growth retardation	86 (14.9)	22 (20.0)	45 (22.5)	19 (7.7)	<0.0001
Enuresis	13 (2.2)	2 (1.8)	7 (3.5)	4 (1.5)	0.33
Frequent URI	125 (21.6)	30 (27.3)	51 (25.5)	44 (16.4)	0.017
Others	23 (4.0)	3 (2.7)	12 (6.0)	8 (3.0)	0.19

^a^Developmental disorder other than CP

Three groups, which were formed based on the use of CAM and other rehabilitation therapies, exhibited a difference in the mean value of disease duration (*P* = 0.015); the group that received neither CAM nor other rehabilitation therapies had the shortest disease duration (55.5 ± 49.4 months). When the patients were divided into two groups according to the median duration of disease (48 months), the current use of CAM and disease duration were significantly related (*P* < 0.0001). In the group receiving CAM or other rehabilitation therapies, the ratio of disease duration over 48 months was 74.4 and 75.8 % each, whereas the group that was receiving neither treatment approach had a ratio of 53.8 %.

Health problems other than the primarily diagnosed disease were related to the use of CAM (*P* = 0.016). The group that was receiving neither CAM nor other rehabilitation therapies had a significantly smaller number of health problems than the other groups (*P* = 0.007). Among the health problems, growth retardation and frequent colds were associated with the use of CAM (*P* < 0.0001 and *P* = 0.017, respectively).

A multinomial logistic regression analysis was conducted to identify the crude OR for CAM use (Table 3). In boys, the OR of CAM use was found to be 0.84 [95 % confidence interval (CI): 0.54–1.30]. In the boys who were not using CAM, the OR of receiving other rehabilitation therapies was 1.45 times (95 % CI: 1.00–2.12) higher than that of the girls. In the patients who were 1–6 years of age, the odds of using CAM was 2.48 times (95 % CI: 1.32–4.65 times) higher than that of those aged 13–19 years. In addition, among the patients who were not using CAM, those who were 1–6 years of age had a odds of receiving other rehabilitation therapies that was 4.64 times (95 % CI: 2.78–7.74 times) higher than that of those aged 13–19 years. For patients with a disease duration equal to or more than 48 months, the odds of using CAM was 2.50 times (95 % CI: 1.28–4.50) higher than that of those with a disease duration of less than 48 months (*P* < 0.001). The OR of using CAM in those who had health problems was 1.38 (95 % CI: 0.88–2.17).

**Table 3:** Crude ORs of current CAM use according to univariate multinomial logistic regression

	Group*		
Variable	CAM user OR (95 % CI)	CAM non-user with other rehabilitation therapies OR (95 % CI)	*P* value**
Sex			
Male	0.84 (0.54–1.30)	1.45 (1.00–2.12)	0.042
Female	1.00	1.00	
Age			
1–6 years	2.48 (1.32–4.65)	4.64 (2.78–7.74)	<0.0001
7–12 years	2.47 (1.38–4.42)	2.10 (1.26–3.50)	
13–19 years	1.00	1.00	
Disease Duration			
< 48 months	1.00	1.00	<0.0001
≥ 48 months	2.50 (1.38–4.50)	2.70 (1.68–4.34)	
Having Health Problems			
Yes	1.38 (0.88–2.17)	1.72 (1.18–2.52)	0.016
No	1.00	1.00	

*Reference category: CAM non-user without other rehabilitation therapies

**Estimated P value of the association between the study variables and the polytomous outcome using the likelihood ratio test

Furthermore, a multivariate multinomial logistic regression analysis was performed to identify the adjusted OR for CAM use (Table 4). Compared with the children who were 13–19 years old, those who were 1–6 years old and those who were 7–12 years old had more frequent use of CAM by 3.14-fold (95 % CI:1.31–7.53) and 3.34-fold (95 % CI:1.64–6.79), respectively. The patients who had a disease duration equal to or more than 48 months had a 3.36 times (95 % CI: 1.71–6.59) higher utilization rate of CAM than that of those who had less than a 48-month disease duration. In the other rehabilitation therapies group, those aged 1–6 years and 7–12 years were 6.92 times (95 % CI: 3.40–14.1) and 2.31 times (95 % CI: 1.36–4.22) more likely to use CAM than those aged 13–19 years, respectively. Moreover, patients who had a disease duration equal to or more than 48 months exhibited a 5.58 times (95 % CI: 3.06–10.2) higher utilization rate of other rehabilitation therapies.

**Table 4:** Adjusted ORs of current CAM use according to multivariate multinomial logistic regression

	Group*		
Variable	CAM user OR (95 % CI)	CAM non-user with other rehabilitation therapies OR (95 % CI)	*P* value**
Sex			
Male	0.85 (0.49–1.49)	1.56 (0.96–2.53)	0.067
Female	1.00	1.00	
Age			
1–6 years	3.14 (1.31–7.53)	6.92 (3.40–14.1)	<0.0001
7–12 years	3.34 (1.64–6.79)	2.31 (1.26–4.22)	
13–19 years	1.00	1.00	
Disease Duration			
<48 months	1.00	1.00	<0.0001
≥48 months	3.36 (1.71–6.59)	5.58 (3.06–10.2)	
Having Health Problems			
Yes	1.12 (0.63–1.99)	1.44 (0.88–2.37)	0.34
No	1.00	1.00	

*Reference category: CAM non-user without other rehabilitation therapies

**Estimated *P* value of the association between the study variables and the polytomous outcome using the likelihood ratio test

## Discussion

This study separately investigated both the experience with each treatment and the current use of the treatment; information regarding the experience rate and the current utilization rate were thus differentially obtained.

First, focusing on ever having had CAM, the present study revealed that the experience rate for CAM was 51.5 %, which was higher than the rate of experiencing CAM in children who visited a neurologic clinic in Canada (44 %) [11] and lower than that of children with a brain injury in the United Kingdom (UK, 59 %) [25]. Although we were unable to compare these populations directly because the CAM list in each study consisted of different therapies and the disease in each study also differed, approximately one-half of the children with neuropsychiatric diseases generally seemed to have experienced CAM. Focusing on a specific disease, the above-mentioned Canadian study indicated an experience rate of 39 % in epilepsy and 38 % in brain injuries [11], and another study conducted in Canada revealed an experience rate of 35.3 % in CP [26]. These results cannot be directly compared, but the studies found a relatively lower experience rate than our present study, which mainly included patients with epilepsy and CP.

Next, regarding the current utilization rate, our present study revealed a 19.0 % utilization rate at the time the survey was performed (21.9 % in epilepsy, 16.1 % in CP and 11.8 % in those with a developmental disorder). Studies on the current use are limited, but a study conducted in the UK found a use rate of 9.4 % in CP [27], which was lower than the result of our present study.

This distinction can be identified in the utilization rate according to each CAM therapy. Our study revealed a current utilization rate of 12.5 % for dietary supplements, 4.1 % for traditional Korean herbal medicine, and 2.2 % for acupuncture. Comparing with studies that were performed with similar diseases, Soo’s study [11] in children who visited a pediatric neurologic clinic indicated that 15 % used chiropractic manipulations, 12 % used dietary therapy, 8 % used herbal remedies, 8 % used homeopathy and 8 % used prayer/faith healing. Cheshire’s study [25] of children with a brain injury revealed that 22.4 % used massage, 21.4 % used osteopath/cranial osteopathy, 18.4 % used aromatherapy, 15.3 % used omega-3 and omega-6 oil supplements and 14.3 % used homeopathy. In Korea, children with neuropsychiatric disease were using a relatively limited set of CAM therapies, whereas in western countries, patients seemed to be using an even distribution of more varied CAM therapies.

We analyzed the patient characteristics in each CAM group, the other rehabilitation therapies group and the non-use group, and the results revealed a longer disease duration in both the CAM group and the other rehabilitation therapies group. There were also more cases of having other health problems in these groups. Among the other health problems, the presence of common upper respiratory infections was the most frequent (27.3 %), and growth retardation was the second most common problem (20.0 %) in CAM group.

Additionally, the mean ages of the three groups were significantly different, and this difference was still statistically significant according to the adjusted ORs. In both the CAM and other rehabilitation therapies groups, those aged 13–19 years used the fewest treatments. In the CAM group, those aged 7–12 years used the most, with an OR of 3.34, followed by those aged 1–6 years with an OR of 3.14. In the other rehabilitation therapies group, those aged 1–6 years used the most therapies, with an OR of 6.92, followed by those aged 7–12 years with an OR of 2.31. This result suggests that parents tend to seek rehabilitation therapies as much as possible at an early stage, and over time, some of the guardians move to CAM treatments or others stop both types of treatment. Hurvitz’s study [18] on CP children indicated that the younger the patient, the higher the utilization rate of CAM, which does not correspond with our results. This discordance might be caused by the variation in the target diseases and types of CAM investigated and by the difference in health care use between the two countries. In the social context of Korea, there is increasing difficulty to continue outpatient treatment as the school year of the patients increases; therefore, we classified the patients into three groups: 1–6 years (before entering elementary school), 7–12 years (during elementary school), and 13–19 years (during middle school and high school). Consequently, preschool children were the most prevalent group in the other rehabilitation therapies group, and elementary school children were the most prevalent group in the CAM group. Various CAM therapies are not widely used in Korea as mentioned earlier; thus, parents seem to first encounter other rehabilitation therapies which are more easily accessible, and they seem to use CAM later when starting to seek other additional treatment if the patients’ disease is not resolved and the disease duration increases. This phenomenon corresponds with the results of Sanders’ study [28], which demonstrated that CAM is more frequently used by those with un-treatable diseases than those with treatable diseases. The OR decreased from 6.92 in the 1–6-years-old age group to 2.31 in the 7–12-years-old age group in the other rehabilitation therapies group, but the OR increased from 3.14 at 1–6 years of age to 3.31 at 7–12 years of age, which implies that preschool children visit hospitals and receive rehabilitation therapies frequently, but school-aged children tend to change to food or drug intake, which does not require a healthcare visit.

In the present study, we only investigated disease duration and not disease severity. When the disease duration was divided into two groups of equal to or more than 48 months and lesser than 48 months, patients with longer disease duration exhibited a significantly higher utilization rate in not only the other rehabilitation therapies but also the CAM group. This result corresponds with other previous studies, and it can be interpreted that CAM is more frequently used in chronic or severe diseases. Samdup’s study [26] also showed increased CAM use in those with greater disease severity of CP. Previous studies also revealed that CAM use is greater in those with more seizures in epilepsy patients [27], more severe diseases or degree [29] of disability [22], and greater inability of independent ambulation [18].

A limitation of this study was the lack of considering the socio-economic factors that are known to affect CAM use, including parents’ age, education background, and economic level. Previous studies have shown that parents’ past CAM use experiences affect their children’s current CAM use [18, 25, 28, 30]; thus, it can be inferred that decision makers’ experiences have significant effects on their children’s treatment. In addition to the parents’ past CAM use, their socio-economic factors affect the children’s CAM use, as shown in previous studies. A higher parental education level [18, 22, 25], older father’s age [18], and higher parental socio-economic status [27] were associated with higher CAM use in children. Greater parental agreement with CAM philosophy [25] was also associated with more frequent CAM use compared to those who do not agree.

Another limitation is that the subjects who had several heterogeneous diseases and the severity of disease, which can affect the use of CAM, were not objectively identified.

In addition, only the opinions of the parents and not those of their children were surveyed. However, this study is meaningful because we analyzed children with neuropsychiatric disorders, which have not been investigated in previous studies. We considered their clinical characteristics and the use of other rehabilitation therapies to analyze the factors of CAM use. Further studies are required to confine the survey to one disease with a more detailed division of each subsection that represents the patient characteristics. Further studies should also evaluate various family factors such as the parents’ socio-economic level.

## Conclusion

This study’s aim was to investigate the present condition and influencing factors of CAM use among children with neuropsychiatric disease. Patients aged 1–6 years and 6–12 years are more frequently administered CAM than those aged 13–19 years, whereas other rehabilitation therapies without CAM are preferred by patients aged 1–6 years, followed by patients aged 6–12 years and then by those aged 13–19 years. Patients with long disease durations use CAM more frequently. Furthermore, the main factors influencing CAM use are disease duration and patient age. This study will supply useful data for developing healthcare systems or services for children with neuropsychiatric disease.

### Ethics

This study was approved by the Institutional Review Board of Pusan National University Hospital (PNUH; 2011038).

### Consent

After the Institutional Review Board approved this study, we explained the study to all of the parents and caregivers, and those who signed the informed consent form were included.

### Availability of data and materials

The data from this trial will be accessible by contacting the corresponding author.

## Additional file

Additional file 1: Questionaire. (DOCX 20 kb)

## Abbreviations

ANOVA: analysis of variance
CAM: complementary and alternative medicine
CI: confidence interval
CP: cerebral palsy
OR: odds ratio
