# Supplementary material for: Current utilization and influencing factors of complementary and alternative medicine among children with neuropsychiatric disease: a cross-sectional survey in Korea
Source: BMC Complement Altern Med. 2016 Mar 1;16:91. doi: 10.1186/s12906-016-1066-4 (PMC4774171; doi:10.1186/s12906-016-1066-4)
Supplement: Additional file 1: — Questionaire. (DOCX 20 kb) [file 12906_2016_1066_MOESM1_ESM.docx]

1. Patient’s gender

□ male □ female

2. The birth of patient

Year: Month: Date:

3. Primary diagnosis

□ epilepsy □ cerebral palsy □ developmental disorder

□ attention deficit hyperactivity disorder (ADHD) □ Tic disorder/Tourette syndrome

□ depressive disorder/obsessive compulsive disorder □ autism disorder

□ intellectual disability □ brain tumor □ head injury □ learning disorder

□ others:( )

4. The onset of the checked disease

Year: Month:

5. The first-visit medical center for the checked disease

□ university hospital / general hospital □ hospital □ traditionalKorean medicine hospital

□ clinic □ traditional Korean medicine clinic

Name of the medical center ( )

6. The medical center in which the checked disease has been diagnosed

□ university hospital / general hospital □ hospital □ traditional Korean medicine hospital

□ clinic □ traditional Korean medicineclinic

Name of the medical center ( )

□ not yet diagnosed

7. The diagnosis date of the above-checked disease

Year: Month:

8. Health problems other than the diagnosed disease (multiple responses possible)

□ atopic dermatitis □ allergic rhinitis □ asthma □ dyspepsia □ growth retardation

□ enuresis □ frequent upper respiratory infection □ others ( )

9. Concurrent therapies **currently in use** (multiple responses possible)

* Concurrent therapies: every therapy for not only the checked disease but also any other diseases, from medical centers other than this hospital

□ none

□ yes

□ speech therapy □ art, music, play, sensory integrative therapy

□ occupational therapy, physical therapy

□ psychological counseling, cognitive behavioral therapy

□ neuro-feedback □ Tuina, massage, acupressure therapy

□ acupuncture/ moxibustion therapy □ traditional herbal medicine

□ dietary supplements

□ Others :( )

10. Concurrent therapies **used in the past** only, not currently in use (multiple responses possible)

* Concurrent therapies: every therapy for not only the checked disease but also any other diseases, from medical centers other than this hospital

□ none

□ yes

□ speech therapy □ art, music, play, Sensory integrative therapy

□ occupational therapy, physical therapy

□ psychological counseling, cognitive behavioral therapy

□ neuro-feedback □ Tuina, massage, acupressure therapy

□ acupuncture/ moxibustion therapy □ traditional herbal medicine

□ dietary supplements

□ Others :( )

11. Concurrent therapies **planning to or willing to use in the future**, not currently in use (multiple responses possible)

* Concurrent therapies: every therapy for not only the checked disease but also any other diseases, from medical centers other than this hospital

□ none

□ yes

□ speech therapy □ art, music, play, Sensory integrative therapy

□ occupational therapy, physical therapy

□ psychological counseling, cognitive behavioral therapy

□ neuro-feedback □ Tuina, massage, acupressure therapy

□ acupuncture/ moxibustion therapy □ traditional herbal medicine

□ dietary supplements

□ Others :( )

12. Please check all of the treatments currently in use in this hospital.

□none

□ yes

□ medication □ speech therapy □occupational therapy, physical therapy

□ psychological counseling, cognitive behavioral therapy
